# Supplementary material for: Yiai Fuzheng decoction inhibits triple-negative breast cancer by remodeling the immune microenvironment
Source: Front Immunol. 2025 Sep 30;16:1615631. doi: 10.3389/fimmu.2025.1615631 (PMC12518410; doi:10.3389/fimmu.2025.1615631)
Supplement: Supplementary file 2 [file DataSheet1.zip › Supplementary File 2/Treatment/D_vs_H/01_Basic_Statistics/Metabolite_Class_Statistics/Class_Pieplot.pdf]

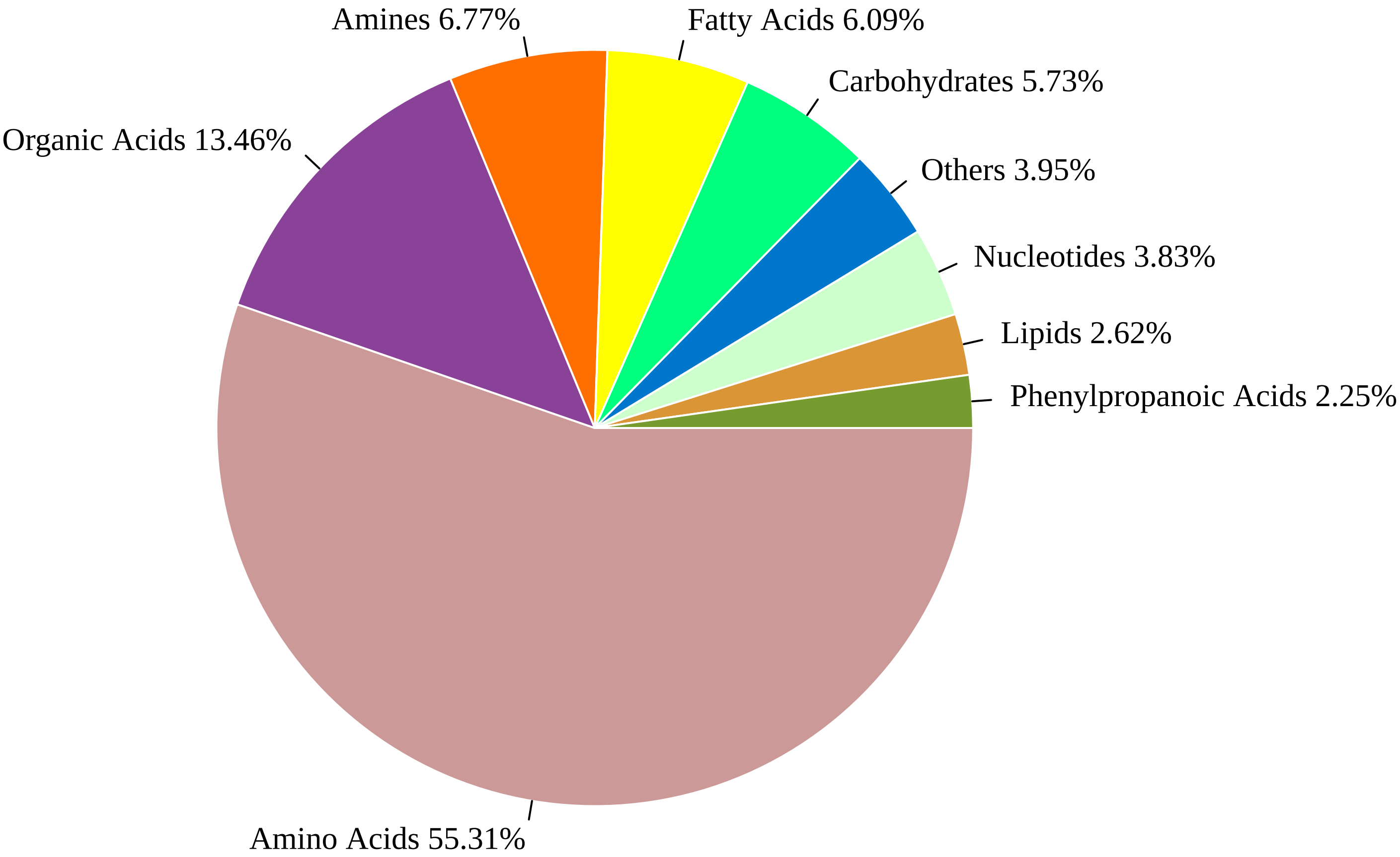
Amines 6.77%
Fatty Acids 6.09%
Carbohydrates 5.73%
Organic Acids 13.46%
Others 3.95%
Nucleotides 3.83%
Lipids 2.62%
Phenylpropanoic Acids 2.25%
Amino Acids 55.31%
